# Breathing Out Completely Before Inhalation: The Most Problematic Step in Application Technique in Patients With Non-Mild Chronic Obstructive Pulmonary Disease

**DOI:** 10.3389/fphar.2019.00241

**Published:** 2019-03-12

**Authors:** Magda Vytrisalova, Tereza Hendrychova, Tereza Touskova, Eva Zimcikova, Jiri Vlcek, Libor Nevoranek, Michal Svoboda, Karel Hejduk, Kristian Brat, Marek Plutinsky, Barbora Novotna, Pavlina Musilova, Matej Cernohorsky, Vladimir Koblizek

**Affiliations:** ^1^Department of Social and Clinical Pharmacy, Faculty of Pharmacy in Hradec Králové, Charles University, Hradec Králové, Czechia; ^2^Department of Pneumology, Faculty of Medicine in Hradec Králové and University Hospital Hradec Králové, Charles University, Hradec Králové, Czechia; ^3^Faculty of Medicine, Institute of Biostatistics and Analyses of the Faculty of Medicine, Masaryk University, Brno, Czechia; ^4^Institute of Biostatistics and Analyses, Ltd., Brno, Czechia; ^5^Department of Pulmonary Diseases and Tuberculosis, Faculty of Medicine, University Hospital Brno, Masaryk University, Brno, Czechia; ^6^Department of Pneumology, Bulovka Hospital, Prague, Czechia; ^7^Pulmonary Department, Regional Hospital Jihlava, Czechia

**Keywords:** chronic obstructive pulmonary disease, adherence to application technique, inhalation systems, inhalation adherence, five steps assessment, device mastery, inhaler mishandlings

## Abstract

**Background:** Patient adherence to an inhaled medication application technique (A-ApplT) represents a major health-care issue in patients with chronic obstructive pulmonary disease (COPD). However, there is a lack of studies evaluating this issue thoroughly. The aim of our study was to introduce a universal easy-to-use method of assessing the A-ApplT to chronic medication in moderate to very severe COPD individuals.

**Methods:** The Czech Multicenter Research Database of COPD (COPD CMRD), a large observational prospective study, was used as a source of clinical data. A-ApplT was evaluated using our Five Steps Assessment. This measure is based on dichotomous evaluation of each of five predefined consecutive application technique steps and can be used in all settings for all currently available inhalation systems in COPD subjects.

**Results:** A total of 546 participants (75.0% men; mean age 66.7 years; mean forced expiratory volume in 1s 44.7%) were available for analysis. This represents 69.6% of all patients recruited in the COPD CMRD. Less than one third of patients presented their application technique without any erroneous steps. The most problematic steps were breathing out completely in one breath immediately before inhalation (step No. 3), and the actual inhalation maneuver (step No. 4). The total number of errors was similar for dry powder inhalers and pressurized metered-dose inhalers.

**Conclusion:** Our novel instrument, Five Steps Assessment, is comfortable for use in routine clinical practice to explore A-ApplT. The A-ApplT in real-life patients with non-mild COPD was inadequate and patients should be repeatedly trained by properly (re-)educated medical staff.

## Introduction

Chronic obstructive pulmonary disease (COPD) is a preventable and treatable progressive lung disease. Its prevalence is increasing worldwide, presenting a substantial medical, social and economic problem (GBD 2015 Chronic Respiratory Disease Collaborators, 2017; Global Strategy for the Diagnosis Management and Prevention of COPD, 2018). COPD is currently the fourth global leading cause of death (WHO Chronic Obstructive Pulmonary Disease, 2017). In the Czech Republic (10.6 million inhabitants), COPD has been reported to be responsible for 21,000 acute hospitalisations and 3,500 deaths annually (Maly et al., 2013).

Although the methods of “evidence-based medicine” have not yet conclusively proven the effect of any existing COPD medication on long-term decline in lung function, pharmacotherapy is fully indicated to reduce symptoms, frequency and severity of exacerbations and to improve exercise tolerance and health status. Currently, inhaled medication represents the cornerstone of COPD pharmacotherapy (Global Strategy for the Diagnosis Management and Prevention of COPD, 2018).

However, as with other chronic diseases, adherence to COPD treatment is often poor. The poor adherence results in increased COPD symptoms, hospitalisations, higher rates of morbidity, healthcare expenditures and reduced quality of life (Bourbeau and Bartlett, 2008; Restrepo et al., 2008; Vestbo et al., 2009; Rogliani et al., 2017). Even though adherence is frequently expressed quantitatively (e.g., percentage of doses used), it is worth noting that non-adherence has principally two forms: (1) a patient uses an incorrect dose of his/her medication and (2) a patient uses his/her medication in an incorrect manner (Boudes, 1998).

In respiratory medicine, however, the “qualitative” aspect is equally important. Incorrect application technique often reduces the effectiveness and can sometimes increase the risks of the used medication. In COPD patients, who are often of higher age and with multiple morbidities, failure to adhere to a specific application technique constitutes a major adherence problem. Inhaler mishandlings can be understood as a type of non-intentional non-adherence sometimes called inhalation non-adherence. We prefer and use the term adherence to application technique or application technique adherence (A-ApplT, also known as device mastery or inhalation adherence/compliance). “Application” is a broader term than “inhalation” and, in addition to the actual inhalation maneuver, includes actions closely associated with the application technique, such as pressing the piercing button, shaking the inhaler etc. For optimal use of an inhaler, it is necessary to manage the application in its entirety. When citing other studies, we use the wording used by their authors.

Studies have shown high rates of inhalation non-adherence and effective use of inhalers in only around 10% of patients (Fink and Rubin, 2005; Lavorini et al., 2008; Restrepo et al., 2008; Laube et al., 2011). Inhaler mishandling is associated with reduced lung drug delivery or deposition and with an increased risk of hospitalisations, emergency room visits, courses of oral corticosteroids and antimicrobials and poor disease control (Fink and Rubin, 2005; Melani et al., 2011). Pharmaceutical industry continues to develop new inhalers with the focus on ease-of-use. In addition, single-inhaler fixed drug combinations may provide greater comfort to patients, particularly those with severe COPD, and thus may secure greater efficacy (Montuschi et al., 2016). Despite the huge effort to make the inhalers more patient-friendly through emphasis on technological aspects and design, errors in an application technique are still frequent in both, pressurized metered dose inhalers (pMDIs) and dry powder inhalers (DPIs) (Melani et al., 2004, 2011; Hammerlein et al., 2011; Rogliani et al., 2017). The use of various inhalation systems and frequent need for combination therapy further complicate the situation in this field (Laube et al., 2011; Global Strategy for the Diagnosis Management and Prevention of COPD, 2018).

At present, there is no methodological “gold standard” as to how to assess A-ApplT. Analyses of erroneous steps in application techniques of various inhalers often use different checklists or different scoring and, therefore, comparison of results is challenging. Furthermore, several studies have assessed A-ApplT in mixed cohorts of patients with various types of respiratory diseases, most frequently with bronchial asthma and COPD. However, there might be substantial differences in cohorts of patients affected by the various diseases in terms of socio-demographic and medical characteristics, leading to discrepancies in their skills and ability to adhere to a particular application technique (Kardas et al., 2013).

Chronic obstructive pulmonary disease management in the Czech Republic is primarily provided at the secondary care level. Unlike Western European countries, the majority of COPD patients in the Central and Eastern Europe are managed by respiratory specialists (Koblizek et al., 2014, 2017). General practitioners (GPs) are the first port of contact for all COPD-risk individuals in the Czech Republic but the COPD diagnosis is almost exclusively confirmed by respiratory specialists. Moreover, vast majority of COPD patients are subsequently managed at respiratory outpatients (in the Czech Republic, there are 330 respiratory specialist outpatients per 10.6 million inhabitants). In addition, it has been reported that A-ApplT in COPD patients varies depending on a clinician’s speciality and their experience (Bourbeau and Bartlett, 2008; Restrepo et al., 2008). Speciality of a physician is also crucial in their own ability to cope with an application technique and the quality of education provided to patients (Melani et al., 2011; Arora et al., 2014). Consequently, our project involved respiratory physicians only and did not include any GPs.

The primary objectives of this study were:

(1) To introduce a universal easy-to-use method and
(2) To assess the rate of adherence to an application technique (A-ApplT) of chronic inhaled medications used in patients with non-mild COPD in routine clinical practice of respiratory physicians.

## Materials and Methods

### Design and Participants

This study provided a baseline evaluation of adherence to an application technique (A-ApplT) within the Czech Multicenter Research Database of COPD (COPD CMRD) (Czech Multicenter Research Database of COPD, 2018). CMRD is an ongoing observational long-term prospective multicenter study with the primary objective of investigating all-cause mortality in patients with non-mild COPD in the Czech Republic, EU (ClinicalTrials.gov NCT01923051: registered 14th August, 2013). COPD patients were recruited to the CMRD from August 2013 to December 2016 by 14 outpatient secondary health care centers providing respiratory physician-based care to patients with COPD. Participation in the COPD CMRD was systematically offered to all consecutive patients who met the inclusion criteria and did not fulfill any exclusion criteria. The study was approved by the Ethics Committee of the University Hospital in Brno (16th January 2013) and then by the Institutional Review Boards and Ethics Committees of all participating centers. Written informed consent was required from all patients.

Inclusion criteria were: diagnosis of COPD, age ≥ 18 years, post-bronchodilator forced expiratory volume in 1s (FEV_1_) ≤ 60 %, stable condition without exacerbations for at least 8 weeks prior to enrolment and a home address in close proximity to the research center. Patients were excluded on the grounds of: cystic fibrosis, terminal stages of a malignancy, end-stage COPD, uncooperative patient or bed-to-chair activity level. Further details on the inclusion and exclusion criteria have been published elsewhere (Novotna et al., 2014).

Within the CMRD study, A-ApplT evaluation was not mandatory but it was highly recommended that this assessment is conducted. Physical examinations, medical records, self-administered instruments and interviews with patients were used to assess socio-demographic and health characteristics (Novotna et al., 2014).

### Outcome Measure

To assess the A-ApplT, five consecutive steps to be followed while using an inhalation system (inhaler) were observed as summarized in Table 1. All types of inhalers currently authorized and used in the treatment of COPD were evaluated (Laube et al., 2011; Czech Multicenter Research Database of COPD, 2018).

**Table 1:** Adherence to application technique (A-ApplT) – a brief description of the five steps for the different groups of inhalation systems used in chronic COPD patients (Five Steps Assessment). (http://chopn.registry.cz/index-en.php).

Step No.	Group of inhalation systems	
	
	Aerosol inhalers (pMDI group)	Dry powder inhalers (DPI group)
**1**	**Getting the inhaler ready for use** (different for different types of inhalers)	
	Remove the mouthpiece cover from the inhaler, and hold the device upright.	Insert a capsule with dry fingers into the chamber (capsule inhalers) and hold the device in correct position
**2**	**Handling the inhaler before use** (different for different types of inhalers)	
	Shake well.	Press the piercing button(s), prepare a dose of drug (non-capsule inhalers).
**3**	**Immediately before inhaling**	
	Breathe out completely in one breath (full and slow exhalation).	
	Do not exhale into the device prior to actuating	
**4**	**Actual inhaling** (different for different types of inhalation devices)	
	While breathing in slowly (4–5 s) and deeply through your mouth, press down on the top of the inhaler with your thumb (press the button) to release a puff	Breathe in quickly and deeply.
**5**	**Immediately after inhaling**	
	Take your inhaler device out of your mouth, hold your breath for several seconds and then breathe out very slowly, away from the inhaler.	Take your inhaler device out of your mouth, hold your breath for several seconds and then breathe out very slowly, away from the inhaler. Inhale twice to empty the capsule completely, close the mouthpiece and clean as needed.


The group of pressurized metered dose inhalers (pMDI group) included three types of inhalers: traditional pMDI (aerosol), EASI–BREATHE and RESPIMAT (soft mist inhaler, SMI). The reasons to include SMI in the pMDI group were as follows: both inhalers are non-DPIs, both require slow and deep breathing in for at least 4 s. In addition, both types of inhalers are the method of choice for patients with low inspiratory flow (Laube et al., 2011). The group of dry powder inhalers (DPI group) comprised of six types of inhalers: HANDIHALER, AEROLIZER (Spinhaler)/BREEZHALER, DISKUS, TURBUHALER (Twisthaler), ELLIPTA, and GENUAIR.

Each patient was asked to carefully demonstrate the use of a placebo inhaler. Patients treated with a combination therapy with two or more different types of inhalers were asked to demonstrate the use of each type. A-ApplT was evaluated and recorded by a nurse under direct supervision (in the same room) of a respiratory physician. The duration of the assessment was up to 5 min in a patient who is treated with 2–3 inhalation systems. Rate of non-adherence to an inhaler was expressed as errors (total score from 0 to 5) at each of the five clearly defined steps (Five Steps Assessment) for each type of an inhaler.

Each step was scored in a simple dichotomous manner: performed correctly (=0) or incorrectly (=1), i.e., used or not used in accordance with the respective manufacturer’s instructions (Summary of Product Characteristics) and European Respiratory Society recommendation (Laube et al., 2011; Czech Multicenter Research Database of COPD, 2018).

Correct performance of the steps for all types of inhalers is elaborated upon in detail in a brief manual available to all participating medical staff (Czech Multicenter Research Database of COPD, 2018). Moreover, all investigators (nurses and respiratory physicians) were trained in the correct use of the Five Steps Assessment before the start of the study (February 2013) and re-trained every year (during annual COPD CMRD working meetings).

The Five Steps Assessment tool was validated by four investigators (authors of this study) who independently scored A-ApplT in a sample of 18 COPD outpatients not included in the present study. The level of agreement among the investigators was pre-set to 90%. The differences in scores between the investigators did not exceed 10% and divergence was always minor in all the tested inhalers.

### Statistical Analysis

Continuous parameters were described with valid N, mean and median (5–95% quantile). Categorical parameters were described with frequencies. Relative frequencies were calculated from valid data. Statistical differences between groups in categorical variables were tested using the Mann-Whitney U test. Relationships between two categorical parameters were analyzed with Fisher’s exact test. The data were analyzed using IBM SPSS Statistics 24.0.0.0. The level of significance was pre-set to α = 0.05.

## Results

Thirteen centers measured adherence to an application technique (A-ApplT) and recruited 546 participants. This represents 69.6% of all patients (*N* = 784) included in the COPD CMRD. The ratio of participants to patients recruited in the COPD CMRD varied from 21.4 to 100.0% per center. Socio-demographic and main clinical characteristics of the participants are summarized in Table 2.

**Table 2:** Socio-demographic and main clinical characteristics of the participants (*N* = 546).

**Basic demography**	
**Age** at entrance into the study in years mean; median (5–95% quantile)	*N* = 546 67.0; 67.0 (51.0–80.0)
**Duration of COPD** from first diagnosis to study (years) mean; median (5–95% quantile)	*N* = 519 8.2; 6.6 (0.5–22.3)
**Men** (%)	408 (75.0)
**Education level:** years spent in pre-graduate school mean; median (5–95% quantile)	*N* = 515 12.0;12.0 (9.0–18.0)
**Smoking status** Ex-smokers (%) Non-smokers (%) Current smokers (%)	386 (71.0) 56 (10.0) 104 (19.0)
**BMI** (kg/m^2^) mean; median (5–95% quantile)	*N* = 546 28.0; 27.0 (18.0–38.0)
**Medical characteristics**	
Moderate and severe exacerbations^∗^ during the last year mean; median (5–95% quantile)	*N* = 546 1.3; 1.0 (0.0; 4.0)
Total number (%) of patients who experienced at least one episode	296 (54.2)
**FEV_1_ (% pred)** mean; median (5–95% quantile)	*N* = 546 44.7; 45.7 (25.1–60.0)
<30 (% pred)	73 (13.4)
30–50 (% pred)	266 (48.7)
>50 (% pred)	207 (37.9)
**FVC (% pred)**	*N* = 546 68.6; 67.9 (39.8–100.7)
**FEV_1_/FVC**	*N* = 546 0.5; 0.5 (0.3–0.7)


*BMI, body mass index; FEV_*1*,_ forced expiratory volume in 1 s; FVC, forced vital capacity. ^∗^deterioration of COPD symptoms which needs treatment with antibiotics and/or corticosteroids (oral or intravenous) during out-patient (moderate) or in-patient (severe) therapy.*

The most frequently used inhalation systems (inhalers) were traditional pMDIs and two types of DPIs: AEROLIZER and HANDIHALER (Table 3). Majority of participants (88%) used a combination of two or more inhalers. The most commonly used dual combinations were: AEROLIZER plus pMDI (*N* = 108; 20.2%) and RESPIMAT plus pMDI (*N* = 22; 4.1%). The most common triple combinations were: HANDIHALER plus AEROLIZER plus pMDI (*N* = 107; 20.0%) and HANDIHALER plus DISKUS plus pMDI (*N* = 59; 11.0%).

**Table 3:** Inhalation systems used by study participants (*N* = 546).

Inhalation system	Participants, N (%)
**Pressurized metered dose inhalers group (pMDI)**	
Traditional pMDI (aerosol)	455 (83.3)
RESPIMAT (soft mist inhaler)	73 (13.4)
EASI - BREATHE	2 (0.4)
**Dry powder inhalers group (DPI)**	
AEROLIZER/BREEZHALER	293 (53.7)
HANDIHALER	240 (43.9)
DISKUS	116 (21.2)
TURBUHALER	40 (7.3)
GENUAIR	21 (3.8)
ELLIPTA	9 (1.6)


**FIGURE 1:**
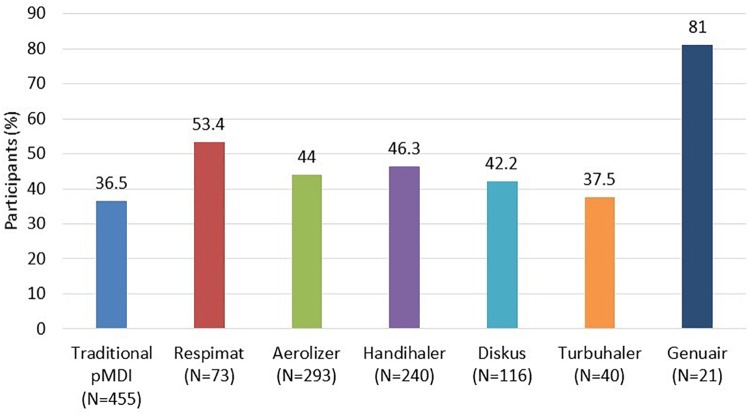
Participants who performed all five steps of the application technique correctly (evaluated by the Five Steps Assessment; total *N* = 546 patients). *N* = number of participants who used the particular inhalation system. Since the majority of participants were treated with a combination of systems, categories are not mutually exclusive. pMDI, pressurized metered dose inhaler. Data for Ellipta and Easy-breathe are not shown, as these devices were used by a small number of participants only (9 Ellipta and 2 Easy-breathe).

Only 164 (30.0%) participants adhered properly to each of the five steps. Full adherence to each type of inhaler, (i.e., all steps performed correctly) is shown in Figure 1.

**FIGURE 2:**
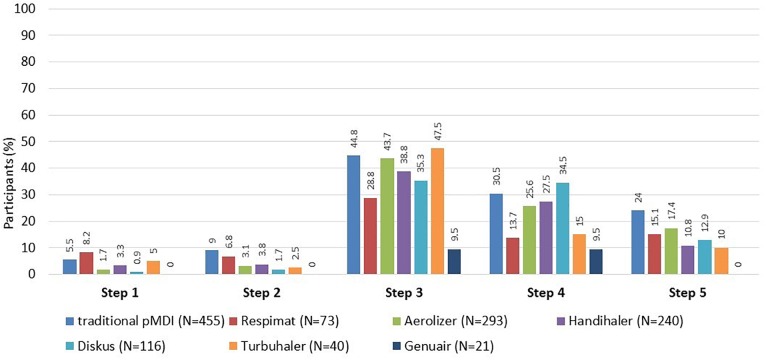
Participants who made error(s) in the application technique (evaluated by the Five Steps Assessment; total *N* = 546 patients). *N* = number of participants who used the particular inhalation system. Since the majority of participants were treated with a combination of systems, categories are not mutually exclusive. pMDI, pressurized metered dose inhaler. Data for Ellipta and Easy-breathe are not shown, as these devices were used by a small number of participants only (9 Ellipta and 2 Easy-breathe).

For all types of inhalers, the highest rate of failure was observed at the step No. 3 (failure to breathe out completely in one breath immediately before inhalation of the drug). The second most problematic step was the step No. 4 (actual inhalation). Erroneous steps for individual types of inhalers are shown in Figure 2.

In patients who used at least one type from each of the two groups of inhalers (DPI and pMDI, *N* = 408), the total number of errors was similar. There was no significant difference between the two groups when comparing the two most erroneous steps (No. 3 and No. 4). Significant differences were observed at the step No. 1 (5.9% in pMDI vs. 3.2% in DPI group, *p* = 0.035), and the step No. 2 (9.3% vs. 3.7%, *p* < 0.001) only.

## Discussion

We have introduced a unique tool for evaluation of adherence to an application technique (A-ApplT) applicable to all types of currently available inhalation systems used in the COPD population. Other studies assessing the A-ApplT (correctness of inhalation) used various types of extensive checklists consisting of a number of different steps relevant to a specific type of inhaler (Molimard et al., 2003; Khassawneh et al., 2008; Rootmensen et al., 2010; Hammerlein et al., 2011; Melani et al., 2011; Pothirat et al., 2015; Price et al., 2017). Our tool is very simple and easy to use. Each step represents a generic type of action, detailed performance of which is specific to the type of inhaler (Table 1). The sequence of these steps is logical and intuitive, allowing facile use in clinical practice and reducing variability if assessed by different clinicians.

Optimal A-ApplT, i.e., application without any error as assessed by our Five Steps Assessment was observed in less than one third (30%) of participants. Pothirat et al. involved a population of COPD patients comparable to ours and identified 25% of patients performing without a critical error (Pothirat et al., 2015). Arora et al. assessed an inhalation technique in patients with asthma and COPD, who were notably younger than our cohort, and found that 82% of participants made at least one error (Arora et al., 2014). Evaluating inhalation techniques in patients affected with asthma and COPD by means of videotaped demonstrations, Rootmensen et al. (2010) concluded that 60% performed all essential steps correctly. However, the cohort of patients observed in the Rootmensen’s study differed substantially from cohorts researched in other studies listed above, including ours. Almost half of their population were patients with asthma in whom bronchial obstruction is reversible and thus patients might feel the effects of their inhalation therapy immediately. Furthermore, their patients had considerably higher mean FEV_1_ (71% predicted) and this can aid the application. On contrary, permanent lung hyperinflation associated with COPD may have fundamental impact on the inhalation technique (Laube et al., 2011).

Since there is no unanimous device to evaluate application techniques, comparison between studies is rather onerous. Nevertheless, available literature agrees on the most problematic steps of application. These are common to pMDIs and DPIs and include: breathing out completely before inhaling (corresponds to our step No. 3), inhaling correctly (step No. 4) and holding breath for several seconds and exhaling away from the inhaler (step No. 5) (Lavorini et al., 2008; Rootmensen et al., 2010; Hammerlein et al., 2011; Arora et al., 2014; Pothirat et al., 2015; Dudvarski Ilic et al., 2016; Bartolo et al., 2017; Price et al., 2017). These observations are in accordance with our findings.

It is clinically important that we observed similar frequency of errors in both groups of inhalers (pMDI and DPI groups) in patients using at least one type from each group. This finding is in accordance with two large studies by Melani et al. (2004, 2011) conducted in routine clinical practice of chest clinics with patients suffering mainly from asthma and COPD as well as with a German study carried out in pharmacies (Hammerlein et al., 2011). On the other hand, Pothirat et al. (2015) and Rootmensen et al. (2010) observed significantly more errors in patients using pMDIs. Molimard et al. (2003) and Khassawneh et al. (2008) made the same conclusions when studying outpatients with asthma and COPD in chest clinics. This may be associated with the different types of inhalers used in the studied cohorts; e.g., single-dose DPIs (Handihaler, Aerolizer), predominant in our study, might be a subject to more mishandlings than prefilled DPIs (Rootmensen et al., 2010). Furthermore, patients who switch to a new drug formulation (e.g., an inhaler recently approved and introduced to the market) can be more able (and willing) to adhere. Patients who are used to a certain type of an inhaler might be less responsive to treatment changes and a different application technique may be more difficult for them to adopt. This could also explain better A-ApplT of GENUAIR.

Incorrect performance of steps No. 1 and No. 2 was infrequent but significantly different between the pMDI and DPI groups. Even though manipulation of single-dose DPIs could be considered more difficult as it requires insertion of a capsule into the inhaler, the pMDI group was associated with more step No. 1 errors. This is probably due to frequently observed inappropriate grasp and positioning of the inhaler. The difference in error rate at the step No. 2 is most likely due to the need to shake the pMDI device. Patients might not consider this to be an important action as the actuation of the device follows. Other authors who focussed on pMDIs mishandling also showed that shaking of the device is one of the most problematic steps (Hammerlein et al., 2011; Pothirat et al., 2015; Bartolo et al., 2017).

No study participant used the spacer device as a tool to facilitate inhalation. In the Czech Republic, spacer devices are only used as an aid in end-stage patients and patients with moderate or severe exacerbations. However, these patients were excluded from the COPD CMRD (Novotna et al., 2014). Consequently, our cohort included patients with stabilized COPD only, in whom spacers are not used.

When we observed and reviewed the A-ApplT, we noticed that many patients breathe out insufficiently before breathing in through the inhaler (step No. 3). In addition, patients were frequently unable to correctly breathe in through the inhaler, e.g., their breathing in was too short or too weak (step No. 4). Therefore, it was necessary to provide patients with a training on the correct application technique. However, it cannot be assumed that all healthcare professionals are fully familiar with the application techniques for the various inhalers. To support health care professionals in their ability to train their patients, Murphy (2019) developed the 7-Steps to Success Inhaler Reminder Cards. We trained the participating respiratory physicians and nurses at workshops during COPD CMRD annual meetings.

If an incorrect breathing pattern is present during inhalation, respiratory physiotherapy techniques can be added to comprehensive treatment. It is possible to use breathing retraining, diaphragmatic breathing, respiratory muscle training, pursed lip breathing and thoracic expansion exercises focused on expansion of lower chest with the aim to improve chest mobility, increase inspiratory and expiratory muscle strength and improve patient’s control of breathing. It is also very important to teach patients correct body positioning during an inhalation. The patient should be in a comfortable well-supported sitting position with his/her back straight and with relaxed upper chest and shoulders (Pryor and Prasad, 2008).

### Strengths

Our study is strong in its use of a large homogenous cohort of patients with moderate to very severe COPD. All centers were secondary care pulmonary outpatient clinics, i.e., the evaluation of A-ApplT was conducted in a consistent manner by trained respiratory nurses under direct supervision of physicians.

Furthermore, our study included all types of inhalers currently used in COPD patients in the Czech Republic. In patients using more than one type of an inhaler, all inhalers were assessed. This provided a comprehensive picture on the patient’s A-ApplT.

### Limitations

Assessments performed within the COPD CMRD within the COPD CMRD were categorized into mandatory and recommended (non-mandatory) ones. The evaluation of A-ApplT is recommended, not mandatory. Data on patients in whom mandatory data were not obtained are considered invalid and not included in analyses (Novotna et al., 2014). Patients who refused participation in the study might be less motivated with poorer health status compared to those who agreed to take part. Therefore, even lower A-ApplT can be expected in the entire real-life COPD population.

The assessment of A-ApplT was subjective, especially with respect to the steps involving exhalation and inhalation. In the absence of equipment to measure these objectively in routine clinical practice, we attempted to minimize the effect of subjectivity by providing unified training (and re-training) in the application technique and handling of each type of the inhaler to all participating nurses and physicians before the study.

It is worth noting that not all inhaler mishandlings reduce lung drug delivery or deposition. However, some of the errors (e.g., failing to coordinate actuation with the start of inspiration in pMDIs) could be critical (Fink and Rubin, 2005; Melani et al., 2011; Price et al., 2017). We did not study clinical impact of the different errors. Although some steps could be more important than others, the main aim still is to manage the application technique in its entirety.

Duration of inhaler use may also have an impact on A-ApplT (Arora et al., 2014). However, we did not measure this. We also did not know how many times a patient was trained in the application technique before entering the study. According to the national COPD guidelines (Koblizek et al., 2013), education should be a part of each patient visit but this is known not to be observed in clinical practice. Patients are properly educated only at the time of diagnosis, at the start of therapy or when it is modified, or when there is worsening of their health status. Finally, it has been suggested that the medication itself could affect A-ApplT (Restrepo et al., 2008; Darba et al., 2015; Koehorst-ter Huurne et al., 2016) but we did not focus on this in our analysis.

## Conclusion

In summary, we developed and validated a unique, easy-to-use instrument, the Five Steps Assessment, which is applicable for evaluation of A-ApplT of all currently available inhalation systems. Our study has shown that the A-ApplT in patients with non-mild COPD is inadequate; only one third of our participants performed all five steps correctly. No significant differences were found between the pMDI and DPI groups. The most problematic steps were breathing out completely before inhalation (step No. 3) and actual inhalation (step No. 4). Therefore, application technique should be repeatedly trained with a focus on the most problematic steps. The training of correct application technique should be performed by properly (re-)educated medical staff.

## Data Availability

The datasets used and/or analyzed during the present study are available from the corresponding author on reasonable request.

## Author Contributions

MV presented the idea of the Five Steps Assessment, participated in data analysis and interpretation, and drafted the manuscript. TH participated in data analysis and interpretation, drafted the manuscript. TT participated in data visualization and interpretation. EZ participated in data interpretation, edited the manuscript. JV contributed to the conception of the manuscript. MS and KH performed data analysis and interpretation. LN, KB, MP, BN, PM, MC, and VK collected the data and supervised adherence evaluation. VK and KH organized the COPD CMRD. MS and VK contributed to writing of the manuscript. VK contributed significantly to data interpretation. All authors read and approved the final manuscript.

## Conflict of Interest Statement

MP received payments on COPD lectures from Boehringer Ingelheim. VK received COPD research funding from Boehringer Ingelheim and Novartis, and received consulting/lectures payments from Angelini, AstraZeneca, Berlin-Chemie, Boehringer Ingelheim, GSK, Mundipharma, Novartis and Sandoz pertaining to the COPD field within the past 3 years. MV, TH, TT, EZ, JV, MS, KH, LN, KB, BN, PM, and MC have not received any payments within the past 3 years. The authors report no other conflicts of interest in this research.

## Abbreviations

A-ApplT: adherence to application technique
CMDR: The Czech Multicenter Research Database
DPIs: dry powder inhalers
pMDIs: pressurized metered dose inhalers
